# Successful treatment with docetaxel plus nintedanib in a patient with lung adenocarcinoma and pulmonary fibrosis: A case report and literature review

**DOI:** 10.3389/fonc.2022.907321

**Published:** 2022-08-09

**Authors:** Tanya Zlatanova, Jeliazko Arabadjiev, Galina Kirova-Nedyalkova, Diana Nikova

**Affiliations:** ^1^ Department of Medical Oncology, Acibadem City Clinic Tokuda Hospital, Sofia, Bulgaria; ^2^ Clinic of Radiology, Acibadem City Clinic Tokuda Hospital, Sofia, Bulgaria; ^3^ Clinic of Pneumology, Acibadem City Clinic Tokuda Hospital, Sofia, Bulgaria

**Keywords:** lung carcinoma, idiopathic pulmonary fibrosis, nintedanib, docetaxel, case report

## Abstract

Despite the rare incidence of idiopathic pulmonary fibrosis (IPF), coexisting IPF and lung cancer is common. Both diseases have unfavorable outcomes and are often associated with impaired quality of life. In this study, we present a clinical case of a patient with coexisting IPF and lung adenocarcinoma who was successfully treated with nintedanib plus docetaxel as a second-line treatment, and achieved a substantial improvement in the quality of life. To our knowledge, very few cases in the literature address the concurrent treatment of both diseases, which makes this case a valuable illustration of a successful treatment strategy and a basis for future investigations.

## Introduction

Idiopathic pulmonary fibrosis (IPF) is a chronic, progressive, fibrotic, and interstitial lung disease with an unknown etiology. IPF often has characteristic imaging and histological appearances that occur primarily in older adults (1). The incidence of IPF is increasing worldwide (2). Similarly, lung cancer is the leading cause of cancer-related mortality worldwide, and its incidence remains very high. According to GLOBOCAN, lung cancer is the second most commonly diagnosed cancer after female breast cancer, accounting for 11.4% of all new cancer cases in 2020 (3). It has been established that IPF patients have an increased risk of developing lung cancer during their lifetime (4). Smoking was evident in the history of both IPF and lung cancer patients. Not only was emphysema found to be common in both IPF and lung cancer patients, but also several common molecular mechanisms have been recognized in both diseases (5). Nintedanib is a tyrosine kinase inhibitor that targets the fibroblast growth factor receptor, vascular endothelial growth factor receptor, and platelet-derived growth factor receptor. Although it was initially discovered as an anticancer agent, nintedanib is a novel therapeutic agent for the treatment of IPF as it slows the disease progression (6). In addition, the combination of nintedanib and docetaxel has proven to be effective for the treatment of advanced non-small cell lung cancer (NSCLC) patients, especially adenocarcinoma patients previously treated with one line of platinum-based therapy. This combination therapy significantly improved progression-free survival and overall survival in those patients (7).

We used the PubMed database to identify four clinical cases in the literature which reported the use of nintedanib for patients having both IPF and lung cancer. Here, we present a clinical scenario that differs from the previously reported clinical cases. This case report presents positive treatment outcomes from the combination of nintedanib and docetaxel as a second-line treatment approach for advanced NSCLC and IPF.

## Symptoms at presentation

A 54-year-old man presented to the clinic with enlarged bilateral neck and right axillary lymph nodes, complaining of high temperature (38 °C) and fatigue. He received ambulatory treatment with the antibiotic cefixime for one week; there was no significant improvement in the enlarged lymph nodes. Upon initial clinical evaluation, he had dense, slightly movable, and painless bilateral neck and supraclavicular lymph nodes together with enlarged bilateral axillary lymph nodes and bilateral inguinal lymphadenopathy. He had an Eastern Cooperative Oncology Group performance status score of 1. His laboratory findings were within the normal reference limits.

The patient was a former smoker with a 40 pack-year smoking history. He quit smoking 15 years ago and worked as a woodworker and builder for the past 20 years. He is currently working as a driver. The patient had mild, well-controlled hypertension and had no reported family history of cancer.

### Physical examination

**Figure 1:**
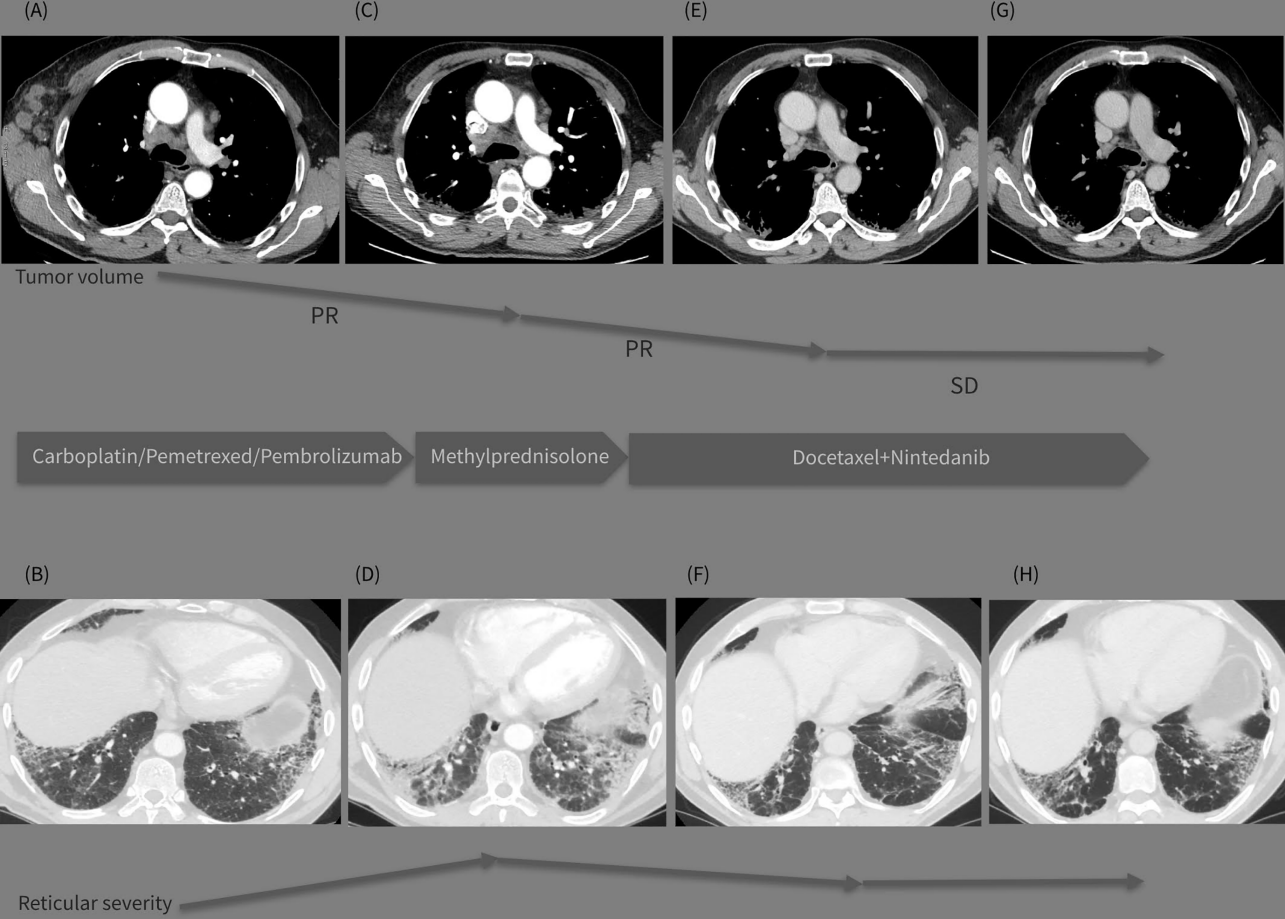
**(A–H)** Graphic summary of the case. The top line presents sequential CT images on mediastinal window **(A, C, E, G)** demonstrating the tumor volume regression during the treatment period (Dec 2020-May 2021-Sept 2021-Dec 2021). The bottom line presents images on lung window **(B, D, F, H)** from the same CT examinations demonstrating the evolution of the interstitial lung abnormalities in the course of the treatment strategy changes (Dec.2020-May 2021-Sept 2021-Dec 2021).

Initial chest computed tomography (CT) (performed on 12/29/2020; **Figures 1A, B**) revealed bilateral supraclavicular, right axillary, and mediastinal lymphadenopathy. It also showed bilateral interstitial lung parenchymal changes, which are characteristic of a fibrotic-phenotype interstitial lung disease. Despite these findings, the patient did not present with typical complaints of unexplained exertional dyspnea or chronic dry cough. Additional invasive procedures including lung biopsy for IPF were proposed but they were refused as the patient was eager to start treatment as soon as possible. At this stage, he did not consult a pulmonologist.

This case posed a diagnostic challenge, and lymphoproliferative disease was initially suspected. After consultation with a hematologist, the patient was admitted to the thoracic surgery ward for a biopsy. A right cervicotomy was performed, and an enlarged right cervical lymph node was excised.

The histology report revealed that the biopsy was a metastatic mass from lung adenocarcinoma. The tumor was CK7^+^, TTF^+^, p63^+^, ALK^+^ without translocation. It had a wild-type EGFR and PD-L1 levels of 42–45%.

The patient underwent positron emission tomography (PET)/CT, which revealed lung carcinoma and multiple enlarged lymph nodes. Initial magnetic resonance imaging (MRI) of the brain did not reveal brain metastases. The final diagnosis was cT1N3M1 lung adenocarcinoma according to the staging manual of the 8^th^ edition American Joint Committee on Cancer, with axillary lymph nodes and solitary muscle metastases. However, the tumor lacked driver gene mutations that could be used for targeted therapy. The multidisciplinary tumor board decided to propose first-line treatment with carboplatin area under the curve (AUC) 5 mg/mL per min, pemetrexed 500 mg/m² and a 200 mg fixed dose pembrolizulab. The patient completed four cycles of this treatment regimen. No grade 3–4 toxicity was observed; however, a grade 1 skin rash and pruritus, classified according to the Common Terminology Criteria for Adverse Events version 5.0, were reported after cycle 1. These complaints resumed after the oral administration of antiallersin.

CT reevaluation (performed on 05/21/2021; **Figures 1C, D**) revealed a partial regression in the number and dimensions of the mediastinal, axillary, and supraclavicular lymph nodes. A substantial progression of the interstitial lung disease with lung fibrosis was observed. This could be likely attributed to the immune and chemotherapeutic treatment received, as autoimmune pneumonitis is one of the typical events of toxicity related to treatment with immune checkpoint inhibitors. The patient reported fatigue, worsening shortness of breath, and intensive bouts of coughing.

The patient was referred to a pulmonologist for the clinical assessment of his lung function. Physical examination revealed nail clubbing, oxygen saturation of 88%, and bilateral fine “Velcro” crackles upon auscultation. Pulmonary function testing confirmed reduced diffusion capacity and restrictive ventilatory defects (**Table 1**).

**Table 1:** Pulmonary function tests before treatment and during follow-up.

Value	Before treatment (05/27/2021)		Follow- up on treatment (09/24/2021)		Follow- up on treatment (11/18/2021)	
	Absolute value	% predicted value	Absolute value	% predicted value	Absolute value	% predicted value
**DLCO-SB**	4.18 mmol/min/kPa	40.2%	4.43 mmol/ min/kPa	42.7%	-	-
**FVC**	2.90 L	62.6%	3.29 L	71.0%	3.39 L	73.4%
**FEV1**	2.46 L	67.0%	2.73 L	74.3%	2.79 L	75.8%
**FEV/FVC**	0.86	86,0%	0.83	83.06%	0.82	81.53%
**% Sat.O2**		88%		92%		94%

DLCO-SB, Single-breath diffusing capacity of the lung for carbon monoxide; FVC, Forced vital capacity; FEV1, Forced expiratory volume in 1 second; FEV1/FVC ratio, the ratio of the forced expiratory volume in the first one second to the forced vital capacity of the lungs; Sat.O2, Oxygen saturation.

### Laboratory results

Laboratory results were within the normal limits (CRP 42.3 mg/L; a negative serology for the ANA-profile [18 types of antibodies]; and a negative serology for ANCA-МРО, PR3, GBM, AMA M2). This rejected the clinical suspicion of a systemic connective tissue disease, which is one of the common reasons for interstitial lung disease. Oral corticosteroid treatment with methylprednisolone was initiated at a dose of 1 mg/kg/daily, with slow dose reduction, and was subsequently replaced with prednisone. After 1 month of corticosteroid treatment, the patient reported improvement in his clinical condition, and a reduction in both asthenia and shortness of breath. The therapeutic challenge at this stage was to choose a second-line treatment for this lung cancer patient having an underlying interstitial lung disease, which clinically worsened during treatment with chemotherapy and immune therapy. The tumor board had a multidisciplinary discussion with a radiologist and a pulmonologist concerning the coexisting diseases of the patient – lung adenocarcinoma and pulmonary fibrosis. Nintedanib is active for both lung adenocarcinoma (7) and IPF (6); thus, second-line treatment with 75 mg/m² docetaxel by intravenous infusion on day 1 (as a switch maintenance therapy) plus 200 mg oral nintedanib twice daily on days 2–21 every 3 weeks were administered until unacceptable adverse events or disease progression occurred. The combination of docetaxel and nintedanib has proven to be effective as a second-line treatment for metastatic NSCLC based on the results of the LUME-LUNG 1 study (7).

The patient completed four cycles of docetaxel+nintedanib between July 5 and September 8, 2021. A follow-up CT scan (performed on 09/23/2021; **Figures 1E, F**) showed a positive response for both IPF and lung cancer, with a reduction in the size and number of mediastinal lymph nodes, and a substantial reduction in the consolidation zones with a partial response. The patient was mildly symptomatic, with improvement in the pulmonary function (**Table 1**) and quality of life; his previously reduced physical capacity almost fully restored. He returned to work with no obvious side effects from the combined treatment, except for grade 1 peripheral neuropathy, classified according to the Common Terminology Criteria for Adverse Events version 5.0.

The patient completed four more cycles of docetaxel and nintedanib between October 8 and December 8, 2021. The only mild side effect was a transient itchy rash on the dorsal surface of the palms, which was successfully treated with 0.05% topical betamethasone cream for one week. The patient reported improvement in the quality of life and he continued to work. Worsening of the peripheral neuropathy was not observed.

A follow-up CT (performed on 12/18/2021; **Figures 1G, H**) revealed stable disease, with persistent enlarged right axillary lymph nodes, mediastinal lymph nodes, and interstitial fibrosis type UIP. Lung capacities and O2 saturation were substantially improved (**Table 1**). The patient continued receiving the same treatment with docetaxel and nintedanib. The clinical timeline of the treatments received is shown in **Table 2**.

**Table 2:** Clinical timeline.

Time	Medical examination	Diagnosis and treatment evaluation	Treatment
12.29.2020 02.02.2021	Chest CT PET/CT Genetic testing: EGFR, ALK, PD-L1 Brain MRI	Lung adenocarcinoma - cT1N3M1 (axillary lymph nodes, solitary muscle metastasis) - AJCC 8th edition	Right cervicotomy and lymph node excision
02.13.2021 04.28.2021	CT of chest, abdomen, and pelvis DLCO, FEV1	Lung cancer - PR; Substantial progression of lung fibrosis	4 cycles carboplatin/pemetrexed/pembrolizumab
05.25.2021 06.25.2021	Pulmonary function testing: DLCO, FEV1	Improvement of the pulmonary function	Corticosteroid treatment with methylprednisolone treatment 1 mg/kg with tapering of the dose
07.07.2021 09.10.2021	CT of chest, abdomen, and pelvis Pulmonary function testing: DLCO, FEV1	Lung carcinoma – PR; Pulmonary fibrosis – reduction	4 cycles docetaxel + nintedanib
10.08.2021 12.08.2021	CT of chest, abdomen, and pelvis Pulmonary function testing: DLCO, FEV1	Lung carcinoma – SD; Pulmonary fibrosis – SD	4 cycles docetaxel + nintedanib

CT, Computed tomography; DLCO, diffusing capacity of the lungs for carbon monoxide; FEV1, Forced expiratory volume in 1 second; PR, partial response; SD, stable disease.

## Discussion

Lung cancer remains the leading cause of cancer-related deaths, accounting for almost 1.8 million deaths per year in 2020, according to GLOBOCAN statistics (3). While IPF is a progressive chronic lung disease with a poor prognosis and a median survival of 3.8 years among adults aged ≥ 65 years in the United States (8), its prevalence has increased gradually worldwide (9), and lung cancer among IPF patients varies between 2.7% to 48% in different publications (10). This highlights the need to identify common molecular pathways implicated in the two diseases and identify optimal treatment strategies for patients having both lung cancer and IPF.

A large retrospective study in Greece between 2011 and 2018 focused on the prevalence and treatment of lung cancer in patients with IPF and enrolled 1016 patients with IPF from eight different centers (11). A hundred - and two cases of concomitant lung cancer were identified, and chemotherapy was applied in 36,5% of patients (11).

Despite the abundance of novel treatment modalities for lung cancer in the past 10 years, including immune therapy, targeted therapy, and chemotherapy, there is currently no consensus regarding the best treatment approach for patients with stage IV lung cancer and coexisting IPF. Data on chemotherapy is insufficient, and there is a need to determine the best therapeutic regimen for the treatment of both diseases.

Watanabe et al. investigated the role of docetaxel as a second-line treatment for patients with platinum-refractory advanced NSCLC and coexisting interstitial lung disease (ILD) (12). The authors concluded that docetaxel monotherapy had poor activity and substantial risks when used for the treatment of platinum-resistant NSCLC with IPF (12).

A retrospective, single-center study by Kanaji et al. revealed a negative effect of docetaxel-containing chemotherapeutic regimens on treatment-related acute exacerbations (13). Additionally, patients with advanced NSCLC and IPF have shorter progression-free survival and overall survival and a lower disease control rate than patients without ILD (13).

Pemetrexed, a folate antimetabolite used as a first- and second-line treatment alone or in combination with cisplatin, or in combination with carboplatin and pembrolizumab against locally advanced and metastatic NSCLC, was proven to have substantial pulmonary toxicity. Kato et al. retrospectively investigated the safety and efficacy of pemetrexed monotherapy in NSCLC patients, with or without coexisting ILD (14). The results demonstrated that the incidence of pemetrexed-related pulmonary toxicity was significantly higher in NSCLC patients with ILD than in those without ILD (14).

Another single-center retrospective study investigated the efficacy and safety of three chemotherapy regimens, carboplatin plus paclitaxel, carboplatin plus docetaxel, and vinorelbine, as first-line chemotherapy in a small cohort of Japanese NSCLC patients with coexisting IPF (15). Out of the 21 patients enrolled in that study, 16 received carboplatin plus paclitaxel and registered an overall response rate of 56.3% (n=9) and a disease control rate of 87.5% (n=14) (15).

The phase 3 LUME-Lung 1 study assessed the efficacy and safety of docetaxel plus nintedanib as second-line therapy for NSCLC (7). This multicenter, multinational randomized study allocated patients in a 1:1 ratio to receive 75 mg/m² docetaxel by intravenous infusion on day 1 plus either 200 mg oral nintedanib twice daily or matching placebo on days 2–21, every 3 weeks until unacceptable adverse events or disease progression occurred (7). The progression-free survival was significantly improved in the docetaxel plus nintedanib group compared with the docetaxel plus placebo group (median, 3.4 months; 95% CI, [2.9–3.9] vs 2.7 months [2.6–2.8]; hazard ratio, 0.79; 95% CI, [0.68–0.92], respectively, *p* = 0.0019) (7). Moreover, patients with a histological adenocarcinoma registered an improvement in the overall survival in the docetaxel plus nintedanib group compared with the docetaxel plus placebo group (median, 12.6 months vs 10.3 months; hazard ratio, 0.83; 95% CI, [0.70–0.99], respectively, *p* = 0.0359). However, this study did not evaluate the efficacy and safety of nintedanib in patients with lung cancer and IPF.

The Japanese J-SONIC randomized phase III study investigates the addition of nintedanib to carboplatin and nab-paclitaxel for the treatment of advanced NSCLC and IPF. The study aims to prove that the combination prolongs the interval to acute exacerbation of IPF compared with chemotherapy alone and the results are eagerly awaited (16).

Using a PubMed database search, we identified four publications regarding nintedanib treatment in patients with coexisting IPF and NSCLC (17–20). One patient was an active smoker, while the others were former smokers. All four patients were males, aged 69–82 years, with stage I to IV NSCLC. One of the four patients had an unknown histology, while the others had the histology of squamous cell carcinoma.

In one clinical case, nintedanib was used as a primary treatment for IPF at a dose of 300 mg/day, which was subsequently reduced to 200 mg/day due to moderate deterioration of liver function (19). During follow-up for IPF, an early stage squamous carcinoma of the lung was diagnosed and resected (19). The authors concluded that nintedanib simultaneously prevented the progression of IPF and the associated lung carcinoma because of its diverse mechanisms of action (19).

The other three clinical cases focus on the treatment of unresectable NSCLC and IPF. The clinical case by Shiratory et al. presented a male patient with advanced NSCLC and IPF treated with three subsequent lines of combined chemotherapy, including docetaxel plus ramucirumab as a second-line treatment (17). Due to the progression of lung cancer and worsening of IPF, the patient finally received best supportive care and was treated with nintedanib monotherapy at a dose of 150 mg bid for IPF (17). One month after the initiation of nintedanib, the authors observed a partial reduction in the primary tumor and pleural dissemination without severe adverse events (17).

Kai et al. demonstrated partial remission of stage IV non-small cell carcinoma without exacerbation of IPF in an 82-year-old male patient who was treated with the best supportive care and nintedanib at a dose of 100 mg twice daily, which was subsequently increased to 150 mg twice daily (18).

Finally, in the clinical case presented by Yamakawa et al., the researchers suggested that the addition of nintedanib to immune checkpoint inhibitors might prevent drug-induced pneumonitis or acute exacerbations of IPF (20). In this case, the patient received four lines of anticancer treatment but developed drug-induced pneumonitis from pembrolizumab and atezolizumab. The addition of nintedanib at a dose of 200 mg/day to atezolizumab allowed the re-administration of the immune checkpoint inhibitors without exacerbation of the drug-induced pneumonitis in IPF combined with lung cancer (20).

In this study, we report a clinical case of a male patient with advanced NSCLC and a coexisting, initially asymptomatic IPF. Ideally, exact immunogenic evaluation had to be performed in order to exclude autoimmune causes of interstitial lung fibrosis. We did not test for myositis-associated autoantibodies, although myopathic inflammatory myopathy might have been the reason for the HRCT pattern, despite ANA negativity (21). This is considered to be one of the limitations of our investigation. We administered first-line combined chemoimmunotherapy with carboplatin, pemetrexed, and pembrolizumab at a standard dose. The patient had good hematological tolerability and no serious toxicities. After four cycles of this treatment regimen, worsened pulmonary function, pulmonary insufficiency, and impaired daily activity were observed. This was confirmed by high-resolution CT, which revealed substantial progression of ILD, presented with large zones of subpleural fibrosis and consolidations. In contrast, a reduction in the size and number of multiple lymph nodes was observed. Owing to the clinical, functional, and the high-resolution CT criteria for worsening IPF during chemo-immunotherapy, we decided to stop the combined carboplatin/pemetrexed/pembrolizumab treatment, and administered corticosteroid treatment until clinical improvement was evident. Thereafter, the treatment was continued by administering second-line chemotherapy plus targeted therapy with docetaxel and nintedanib as antifibrotic and antitumor agents. After eight treatment cycles with docetaxel and nintedanib, the patient regained a good quality of life, and a follow-up CT reported stable disease. We also observed stabilization of the ILD, with improved pulmonary function tests and substantial reduction of the consolidation zones. A large international survey among pulmonologists, thoracic surgeons and oncologists focusing on the management of IPF and lung cancer raises the need of a consensus statement for the management of these patients (22). Our clinical case suggests that the combination of docetaxel and nintedanib might be an optimal therapeutic option for patients with lung adenocarcinoma and coexisting IPF. Currently, there is a need to identify the most favorable therapeutic regimen for the treatment of these lung diseases. Further prospective studies with precise patient selection are required.

## Patient perspective

I am happy that doctors all over the world are learning from my case and do not mind my condition being discussed. I do not want anyone to go through what I had to. I am very independent now and look after myself. I feel much better with this treatment and I am capable of doing my everyday activities.

## Data availability statement

The original contributions presented in the study are included in the article/supplementary material. Further inquiries can be directed to the corresponding author.

## Ethics statement

Written informed consent was obtained from the individual(s) for the publication of any potentially identifiable images or data included in this article.

## Author contributions

TZ, JA, DN, and GK-N collected the clinical, diagnostic, and therapeutic data. GK-N collected the images of the patients. TZ identified the case, wrote and submitted the manuscript. JA and GK-N have revised and proofread the manuscript. TZ, JA, GK-N, and DN have equally contributed to the study. All authors have contributed to the manuscript and approved the submitted version.

## Acknowledgments

The authors would like to acknowledge the contributions of Dr. Jordan Kovachev for technical support.

## Conflict of interest

The authors declare that the research was conducted in the absence of any commercial or financial relationships that could be construed as potential conflict of interest.

## Publisher’s note

All claims expressed in this article are solely those of the authors and do not necessarily represent those of their affiliated organizations, or those of the publisher, the editors and the reviewers. Any product that may be evaluated in this article, or claim that may be made by its manufacturer, is not guaranteed or endorsed by the publisher.
